# Molecular evolution under increasing transposable element burden in *Drosophila*: A speed limit on the evolutionary arms race

**DOI:** 10.1186/1471-2148-11-258

**Published:** 2011-09-14

**Authors:** Dean M Castillo, Joshua Chang Mell, Kimberly S Box, Justin P Blumenstiel

**Affiliations:** 1 Department of Ecology and Evolutionary Biology, University of Kansas, Lawrence Kansas 66045, USA; 2 Department of Biology, Indiana University, Bloomington, IN 47405, USA; 3 Life Sciences Centre (Zoology), 2350 Health Sciences Mall, University of British Columbia, Vancouver, BC, V6T 3Z4, Canada

## Abstract

**Background:**

Genome architecture is profoundly influenced by transposable elements (TEs), and natural selection against their harmful effects is a critical factor limiting their spread. Genome defense by the piRNA silencing pathway also plays a crucial role in limiting TE proliferation. How these two forces jointly determine TE abundance is not well understood. To shed light on the nature of factors that predict TE success, we test three distinct hypotheses in the *Drosophila *genus. First, we determine whether TE abundance and relaxed genome-wide purifying selection on protein sequences are positively correlated. This serves to test the hypothesis that variation in TE abundance in the *Drosophila *genus can be explained by the strength of natural selection, relative to drift, acting in parallel against mildly deleterious non-synonymous mutations. Second, we test whether increasing TE abundance is correlated with an increased rate of amino-acid evolution in genes encoding the piRNA machinery, as might be predicted by an evolutionary arms race model. Third, we test whether increasing TE abundance is correlated with greater codon bias in genes of the piRNA machinery. This is predicted if increasing TE abundance selects for increased efficiency in the machinery of genome defense.

**Results:**

Surprisingly, we find neither of the first two hypotheses to be true. Specifically, we found that genome-wide levels of purifying selection, measured by the ratio of non-synonymous to synonymous substitution rates (ω), were greater in species with greater TE abundance. In addition, species with greater TE abundance have greater levels of purifying selection in the piRNA machinery. In contrast, it appears that increasing TE abundance has primarily driven adaptation in the piRNA machinery by increasing codon bias.

**Conclusions:**

These results indicate that within the *Drosophila *genus, a historically reduced strength of selection relative to drift is unlikely to explain patterns of increased TE success across species. Other factors, such as ecological exposure, are likely to contribute to variation in TE abundances within species. Furthermore, constraints on the piRNA machinery may temper the evolutionary arms race that would drive increasing rates of evolution at the amino acid level. In the face of these constraints, selection may act primarily by improving the translational efficiency of the machinery of genome defense through efficient codon usage.

## Background

In sexual species, genetic parasites such as transposable elements (TEs) can proliferate to the detriment of the host [1]. Natural selection is widely considered the dominant force limiting TE proliferation [2-4]. The selective forces limiting TE abundance are thought to act against three primary consequences of TE proliferation- gene mutation by TE insertion, chromosomal rearrangement caused by ectopic recombination among dispersed copies, and the energetic burden imposed on the host arising from the costs of replication, transcription and translation of TE copies. TE insertion alleles often segregate at low frequencies in populations, consistent with natural selection limiting their increase. However, studies in recent years have shown that different modes of RNA silencing also play an important role in constraining TE proliferation [5-7]. In particular, within the germline of animals, the piRNA machinery functions as an immune system to protect the genome against TE proliferation [8-10]. TE copies that have inserted into distinct chromosomal regions known as piRNA clusters are recognized as aberrant and their transcripts are directed to the piRNA biogenesis machinery. In complex with Argonaute proteins, 26 - 31 nt piRNAs use sequence identity to target transcripts of dispersed TE copies for degradation. In turn, this generates secondary piRNAs that feed into the cycle of piRNA biogenesis and TE silencing.

These two forces - natural selection and genome defense by small RNAs - directly limit TE proliferation, but in distinct ways. In particular, natural selection will primarily act to limit the increase of harmful TE insertion alleles. Conversely, the piRNA machinery will act by directly limiting the transposition rate, and thus the rate of production of new insertion alleles. How these forces jointly determine the rate of TE proliferation is poorly understood [11]. In fact, the distinction between natural selection and genome defense as separable forces may be artificial, since the genome defense machinery is itself the product of natural selection.

To what degree does the strength of natural selection explain variation in TE abundance across species? Population genetic theory predicts that the strength of selection relative to genetic drift will be greater in larger populations [12]. Thus, as indicated by Ohta, mildly deleterious substitutions will fix at a greater rate in smaller populations. This prediction has been confirmed in a variety of systems where purifying selection was estimated using the rate ratio of non-synonymous to synonymous substitution (ω). Consistent with natural selection acting more strongly against deleterious non-synonymous substitutions, larger populations tend to have smaller ω values. For example, in mammals there is a strong negative correlation between population size and ω [13]. A large study comparing branch-specific ω between closely related mainland and island species found that ω estimates were higher for island species, likely due to their smaller population size [14]. This was observed in both vertebrates and invertebrates. If variation in TE abundance across species is also influenced by the strength of selection relative to drift, TE abundance and genome-wide ω values for protein-coding genes should be positively correlated. This is because differences in population size will modulate the efficacy of selection against both types of mutation.

This hypothesis, however, relies on several assumptions. First, it assumes that variation in ω is more strongly explained by the influence of mildly deleterious substitutions rather than adaptive substitutions. If beneficial mutations are common and much more likely to fix in larger populations, the negative relationship between ω and population size will be ameliorated. Studies in *Drosophila*, however, have indicated that population size has little effect on the rate of adaptive fixation [15,16]. Second, it assumes that the rates of both forms of mutation are independent of population size. Among related species, this is likely for mutations at the nucleotide level, but may be violated for TE insertions if exposure to TE invasion is greater in larger populations. Finally, it assumes that the distribution of fitness effects is similar between TE insertions and non-synonymous mutations and doesn't consider the non-linear scaling of the probability of fixation of mildly deleterious alleles with effective population size [17]. When the product of the effective population size and deleterious selection coefficient (*N_e_s*) is substantially greater than one, the chance that such a deleterious allele fixes becomes vanishingly small. Only nearly neutral deleterious mutations for which *N_e_s *is less than one are expected to fix. Thus, the degree to which variation in population size governs the accumulation of deleterious alleles, such as TE insertions, itself depends on population size. In organisms with very large population sizes drift becomes extremely weak, and modest variation in population size among related species may only impact the rate of accumulation of very mildly deleterious alleles with selection coefficients very close to zero [18]. In this case, the distribution of fitness effects of mildly deleterious mutations will be an important factor in determining the relationship between population size and ω. Setting aside the effects of beneficial mutations, a negative correlation will be observed across all population sizes only if there are a sufficient number of nearly neutral mutations at all population sizes. If the distribution of fitness effects for deleterious mutations does not display this characteristic, modest increases in population size in very large populations may not always lead to decreased ω. For similar reasons, one might not expect a simple relationship between TE accumulation and population size when population sizes are large.

This latter point is highlighted by studies of TE dynamics yielding contrasting results for different species. In *Drosophila melanogaster*, studies have suggested that the deterministic forces of selection against TEs may greatly outweigh genetic drift as a factor [19,20]. *D. melanogaster *also has a large effective population size. Thus, modest variation in population size among species across the genus *Drosophila *may not be an important factor contributing to variation in TE abundance. In contrast, studies in vertebrates with much smaller effective population sizes have shown that genetic drift can be an important factor contributing to TE accumulation [21]. For example, the frequency distribution of TE insertion alleles in the pufferfish is consistent with neutrality [22]. This indicates that variation in effective population size at these low population sizes may have a greater impact on the fate of mildly deleterious TE insertions, relative to species with larger population sizes such as *Drosophila*. It also indicates that in species with larger population sizes, there may only be a weak correlation between the rate of non-synonymous substitution and TE abundance. This may explain why, in one study, after correcting for phylogenetic signal, no apparent relationship between genomic TE number and population size was found [23]. Considering these issues, we aimed to test the simple hypothesis that TE abundance is positively correlated with genome-wide ω across the *Drosophila *genus. Rejection of this hypothesis would support alternative models and provide further testable hypotheses in the study of TE dynamics in large populations.

An added level of complexity is the evolutionary dynamic between TEs and the piRNA machinery. In several species of *Drosophila*, many, but not all, components of the piRNA machinery show a high rate of adaptive evolution [24-26]. This has been proposed to arise from an evolutionary arms race between the host and TEs. Evolutionary arms races between hosts and parasites drive cycles of adaptation and counter-adaptation, leading to increased rates of adaptive evolution in host immune systems. In the case of TEs, there is likely strong selection to avoid silencing by the piRNA machinery. This may select for functions analogous to those observed in viruses, which have mechanisms that directly antagonize the machinery of RNA silencing [27,28]. Reciprocally, natural selection acting on the host likely selects for changes that counteract these strategies, driving a high rate of adaptive evolution in the proteins of the piRNA machinery. But importantly, the classic evolutionary arms race is not sufficient to explain all evolutionary dynamics between host and parasite [29-31]. For example, trench warfare may occur when a diversity of parasites selects for the maintenance of multiple defense strategies, thus favoring a mode of balancing selection [32]. While an evolutionary arms race with TEs is suggested by the high rate of evolution in the piRNA machinery in some *Drosophila *species, it is not clear that increasing TE abundance could drive ever-increasing levels of amino-acid evolution. Constraint on core function will eventually pose some limit to rates of amino-acid evolution. Additionally, with increasing TE abundance, the balance of forces may begin to favor purifying selection over adaptation. To explore these issues, we test the simple hypothesis that in the *Drosophila *genus, increasing TE abundance drives a higher rate of amino-acid substitution in the piRNA machinery as measured by ω.

In this study, we found that genome-wide levels of purifying selection are greater (smaller ω) in *Drosophila *species with higher TE abundance, inconsistent with a model in which increasing TE abundance and ω are jointly explained by weaker selection relative to drift. Compared to control genes, we also find that this observation is more evident in the piRNA machinery. Strikingly, we find that TE abundance and levels of codon bias are positively correlated in the piRNA machinery but not in control genes or in the rest of the genome. Rather than an increasing rate of amino-acid evolution, the primary response of the piRNA machinery to increasing TE abundance appears to be through improved codon usage for increased translational efficiency.

## Results

### Genome-wide levels of purifying selection and TE abundance are positively correlated

**Figure 1:**
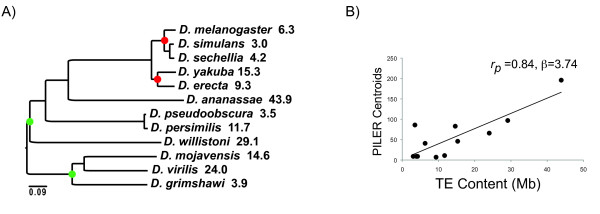
**A) Phylogenetic tree of the *Drosophila *species used in this study, with TE abundance indicated in Mb**. Dots indicate nodes for which ω was estimated on descendant foreground branches. B) TE abundance is correlated with TE family number. Unique, species-specific PILER-DF centroids vs. genomic TE abundance (*p = 0.001*). *r_p_*: Pearson's correlation coefficient. β: regression coefficient (slope).

Using the twelve sequenced *Drosophila *genomes (Figure 1a), we determined the relationship between TE abundance and previous estimates of genome-wide average ω on terminal branches [33]. TE abundance was quantified using the total amount of assembled euchromatin comprised of repeats as determined from the 12 *Drosophila *genomes consortium [34]. This measure captures each of the components of selection considered important against TEs - gene mutation, ectopic recombination and metabolic cost. This measure does ignore the influence of heterochromatic TEs and some species with low euchromatic content may in fact have many TEs within the large domains of pericentric heterochromatin. The converse may also be true. However, it is clear that the gene rich and highly recombining environment of the euchromatin makes insertions in this portion of the genome substantially more harmful. Thus, euchromatic TE content is likely to serve as the best metric for the selective burden of TE content on a species. It is also correlated with the genomic number of TE families (Figure 1b). Using computationally predicted PILER-DF libraries [35] as a measure of TE family number, we find that the number of different TE families within each species' genome is a good predictor of total TE abundance (Figure 1b, p *= 0.001*).

**Figure 2:**
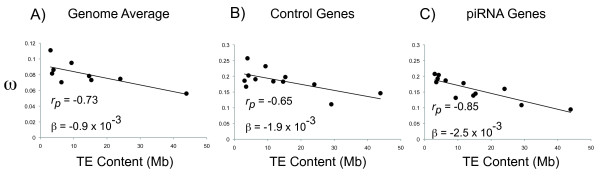
**Genomic TE abundance vs. ω from: A) Genome wide estimates on terminal branches obtained from Heger and Ponting **[33]. Excludes *D. sechellia, D, willistoni *and *D. persimilis *(*p = 0.026*). B) Path ω estimates for control genes. (*p = 0.022*). C) Path ω estimates for piRNA genes (*p < 0.001*). *r_p_*: Pearson's correlation coefficient. β: regression coefficient (slope).

We found a significant negative correlation between genomic TE abundance and tip ω estimates (Figure 2a, p *= 0.025*). When assessing correlations among species, it is essential to account for phylogenetic non-independence [36,37]. This was achieved using the implementation of *Continuous *in the *BayesTraits *package which models evolution of continuous traits on a phylogeny using a generalized least-squares approach under Brownian motion model of evolution. First, we tested whether phylogenetic correction was needed by testing whether the λ scaling parameter significantly deviated from 0 (hereafter designated the λ test). A λ parameter equal to zero indicates trait evolution can be effectively modeled as if it were on a star phylogeny. In this case, a phylogenetic correction was not mandated using a likelihood ratio test (λ test, *p = 0.99*). Nonetheless, we tested whether the correlation was significant with phylogenetic non-independence taken into account. This was performed by testing whether the covariance between the two traits was significantly greater than zero, assuming no scaling transformation of the phylogeny (hereafter designated the *BayesTraits *test). Nonetheless, after accounting for phylogenetic non-independence, the negative correlation between genomic TE abundance and genomic ω estimates remained significant using a likelihood ratio test (*BayesTraits test, p = 0.0078*). To account for potential problems with estimating ω over long terminal branches, we also evaluated the relationship between TE abundance and ω estimates only across the *D. melanogaster *subgroup. Considering only *D. simulans*, *D. melanogaster*, *D. yakuba *and *D. erecta*, there is still a negative relationship between TE abundance and genome-wide ω (*r_pearson _= -0.60, β = -0.0022)*, albeit non-significant with only four data points. Overall, these results indicate that genomes more encumbered by TEs have had a historically greater magnitude of purifying selection acting on protein coding sequences. This is difficult to reconcile with a model in which TE abundance is largely determined by the degree to which mildly deleterious alleles are removed by natural selection within populations.

### Increased purifying selection on the piRNA machinery in species with high TE abundance

Considering the background genome-wide correlation between TE abundance and levels of purifying selection on coding sequences, we next determined how the rate of amino-acid evolution of the piRNA machinery was shaped by TE abundance (Figure 2b and 2c). The rate of molecular evolution for eleven known components of the piRNA machinery and eleven control genes was determined by estimating ω [38] on the evolutionary paths leading to all twelve *Drosophila *species with sequenced genomes. Path estimates of ω were obtained, rather than terminal branch estimates, since terminal branch lengths vary widely between species. Since piRNA genes are known to evolve quickly, a set of control genes was selected, matched for a similar rate of evolution as the piRNA genes. If increasing TE abundance drives a faster arms race, we might expect elevated ω on the piRNA machinery along lineages with greater TE abundance. However, this was not the case. Without phylogenetic correction, control genes (*p = 0.022*) and piRNA genes (*p = 0.0005*) both show a negative relationship between TE abundance and average ω, consistent with the observed genome-wide negative correlation between TE abundance and ω. However, both the *r_pearson _*and β values were more negative for the piRNA genes compared to control genes. Furthermore, for control genes, the λ test indicated that phylogenetic correction was needed *(*λ test, *p = 0.024*) and this correlation was not significant after the correction was made *(BayesTraits, p = 0.243)*. In contrast, even though the λ test indicated no phylogenetic correction was needed for the piRNA genes *(*λ test, *p = 0.439)*, the result was still significant after phylogeny was taken into account (*BayesTraits, p = 0.029*)

**Figure 3:**
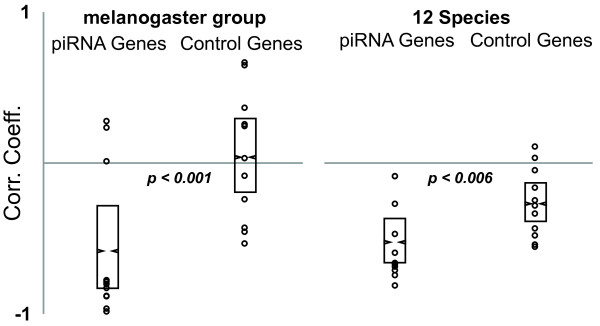
**Distribution of raw Pearson correlation coefficients for the correlation between TE abundance and ω**. Boxes are 95% confidence intervals for the mean.

**Figure 4:**
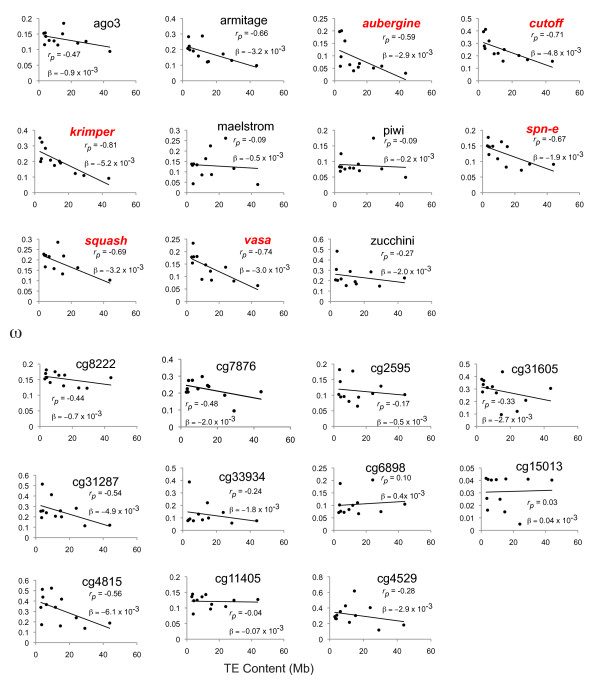
**Genomic TE abundance vs. path ω values for piRNA genes and control genes for all twelve *Drosophila *species**. *r_p_*: Pearson's correlation coefficient. β: regression coefficient (slope).

**Figure 5:**
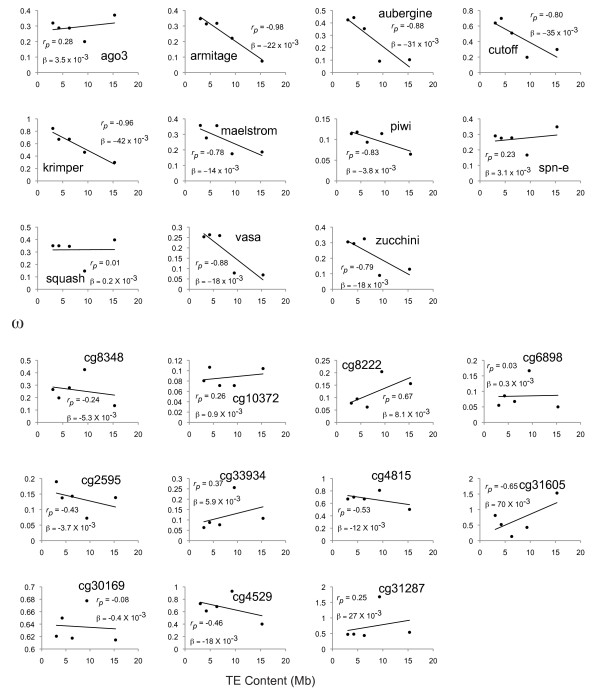
**Genomic TE abundance vs. path ω values for piRNA genes and control genes for the *Drosophila melanogaster *group**. *r_p_*: Pearson's correlation coefficient. β: regression coefficient (slope).

We then sought to determine if this effect was more evident in the piRNA machinery due to artifacts of estimating ω on long branches. We did this by testing whether this trend was also observed in the more closely related *D. melanogaster *group *(D. melanogaster, D. simulans, D. sechellia, D. yakuba *and *D. erecta)*. Focusing only on this set of species, path ω was re-estimated over these shorter divergence times (Figure 1a). With only five species, there is limited power in a single correlation using average values, so we examined the distribution of correlation coefficients between TE abundance and ω in piRNA genes relative to the control gene set (Figures 3, 4 and 5). This served to test whether TE abundance better explained variation in ω for the piRNA genes, compared to control genes, across different levels of divergence. The distribution of correlation coefficients is significantly more negative for piRNA genes than control genes. This is the case in the five species within the *D. melanogaster *group (Bootstrap test for difference in mean, *p < 0.001*) and also all twelve species (Bootstrap test for difference in mean, *p < 0.006*). In addition, to compare effect size, we also tested whether the distribution of β regression coefficients (measures of slope) were different between piRNA genes and control genes. β values were more negative for both the *D. melanogaster *and 12 species comparison. In the case of the *D. melanogaster *comparison, this was significant (Mean β: Control genes, 0.007, piRNA genes, -0.016, *p = 0.016*, t-test). In the case of the 12 species comparison, β was 30% steeper for the piRNA machinery, but this wasn't significant (Mean β: Control genes, -0.0019, piRNA genes, -0.0025, *p = 0.464*, t-test). Thus, we can conclude that in comparison to the control genes, TE abundance better explains variation in ω in the piRNA machinery across different levels of divergence. Moreover, the strength of the effect of TE abundance on ω is especially strong when shorter time scales of divergence are examined.

The above analysis does not account for phylogenetic non-independence. Given the excess of negative correlation coefficients for piRNA genes compared to control genes, we next sought to determine whether this excess would be maintained after accounting for phylogenetic non-independence. With the small sample size of five species in the *melanogaster *group, phylogenetic correction is precluded due to low power [39] and we instead focused solely on all twelve species. After controlling for phylogenetic non-independence where mandated, six of the eleven piRNA genes (Table 1), but none of the control genes (Table 2), show a significant relationship between ω and TE abundance, and this enrichment is significant (Fisher's exact test, 2-tail, *p = 0.012*). Thus, even after accounting for phylogeny, the effect of TE abundance on ω is more evident in the piRNA machinery than for other quickly evolving genes.

**Table 1:** Summary of tests of significance for regression of piRNA gene ω on TE abundance, accounting for phylogenetic non-independence

Gene	LR (for λ)	p-value	*BT* p-value	*R^2^*	p-value
ago3	1.198	0.2737	NA	0.2221	0.1229

armitage	11.417	**7.2759E-4**	0.0623	NA	NA

*aubergine*	2.293	0.1299	NA	0.3521	**0.0419**

*cutoff*	0	1	NA	0.5046	**0.0096**

*krimper*	0	1	NA	0.6579	**0.0014**

maelstrom	9.952	**1.6074E-3**	0.2556	NA	NA

piwi	15.921	**6.6049E-5**	0.5259	NA	NA

*spn-E*	11.119	**8.5444E-4**	**0.0142**	NA	NA

*squash*	3.649	0.0561	NA	0.4684	**0.0141**

*vasa*	4.174	**0.0410**	**0.0165**	NA	NA

zucchini	11.88	**0.0006**	0.0704	NA	NA

Column two: Likelihood ratio value for testing whether phylogenetic signal must be accounted for (λ ≠ 0). Column three: p-value for testing λ = 0. Column four: p-value for testing non-independence using *BayesTraits*. Column five: where phylogenetic corrections were not necessary, the *R^2 ^*for the raw correlation. Column six: p-value for significance of raw regression. Significant p-values are in bold. Genes with a significant effect are italicized.

**Table 2:** Summary of tests of significance for regression of control gene ω on TE abundance, accounting for phylogenetic non-independence

Gene	LR (for λ)	p-value	*BT* p-value	*R^2^*	p-value
CG8222	7.7327	**0.0209**	0.3091	NA	NA

CG7876	1.8889	0.3889	NA	0.2299	0.1147

CG2595	17.1938	**0.0002**	0.4445	NA	NA

CG31605	5.2575	0.07216	NA	0.1121	0.2874

CG31287	6.3365	**0.0421**	0.5858	NA	NA

CG33934	15.5277	**0.0004**	0.5685	NA	NA

CG6898	15.8096	**0.0004**	0.9784	NA	NA

CG15013	19.0398	**7.337E-05**	0.9916	NA	NA

CG4815	0.1274	0.7212	NA	0.3078	0.06118

CG11405	2.8365	0.0921	NA	0.00224	0.8839

CG4259	0	1	NA	0.07935	0.3751

No genes show a significant effect.

### Observed correlation between TE abundance and purifying selection in the piRNA machinery is independent of effects of dS

Variation in ω can be explained by a variety of factors not directly related to the forces of divergence on protein-coding function. For example, lower values of ω in the piRNA machinery of species with greater TE abundance could be explained by an increased rate of silent substitution rather than a decreased rate of amino-acid substitutions. To test this, we determined whether either dN or dS were separately correlated with TE abundance after accounting for phylogenetic non-independence. Considering dN alone, seven piRNA genes show a significant relationship with TE abundance (Table 3). In contrast, only two piRNA genes show a significant relationship between dS and TE abundance (Table 4). Of eleven piRNA genes, only krimper shows a significant relationship between TE abundance and dS, but not dN. Excluding krimper, there is still a significant enrichment in the piRNA genes for a negative relationship between TE abundance and ω (Fisher's exact test, 2 tail, *p = 0.035*). Thus, we can conclude that variation in dS is not sufficient to explain the excess of significant negative correlations between TE abundance and ω in genes of the piRNA machinery.

**Table 3:** Summary of test of significance for regression of piRNA gene dN on TE abundance, accounting for phylogenetic non-independence

Gene	LR (for λ)	p-value	*BT* p-value	*R^2^*	p-value
ago3	4.84815	**0.0276**	0.1241	NA	NA

*armitage*	13.7356	**0.0002**	**0.0068**	NA	NA

*aubergine*	5.38383	**0.0203**	**0.0045**	NA	NA

*cutoff*	5.41623	**0.0200**	**0.0172**	NA	NA

krimper	0.78252	0.3763	NA	0.00327	0.8397

*maelstrom*	9.56345	**0.0020**	**0.0491**	NA	NA

*piwi*	6.51916	**0.0107**	**0.0182**	NA	NA

*spn-E*	13.2482	**0.0003**	**0.0190**	NA	NA

squash	16.3476	**5.272E-05**	0.0742	NA	NA

*vasa*	10.3213	**0.0013**	**0.0031**	NA	NA

zucchini	1.51457	0.2184	NA	0.0001	.5134

Genes with a significant effect are italicized.

**Table 4:** Summary of test of significance for regression of piRNA gene dS on TE abundance, accounting for phylogenetic non-independence

Gene	LR (for λ)	p-value	*BT* p-value	*R^2^*	p-value
ago3	8.8538	**0.0029**	0.1477	NA	NA

armitage	6.6971	**0.0096**	0.961	NA	NA

*aubergine*	10.7708	**0.001**	**0.0369**	NA	NA

cutoff	5.8223	**0.0158**	0.5921	NA	NA

*krimper*	8.9521	**0.0027**	**7.83E-06**	NA	NA

maelstrom	6.0984	**0.0135**	0.4427	NA	NA

piwi	7.0476	**0.0079**	0.0863	NA	NA

spn-E	10.9271	**0.0009**	0.3563	NA	NA

squash	4.059	**0.0439**	0.5131	NA	NA

vasa	10.3282	**0.0013**	0.901	NA	NA

zucchini	5.5428	**0.01859**	0.3007	NA	NA

Genes with a significant effect are italicized.

### Observed correlation between TE abundance and purifying selection in the piRNA machinery is independent of other confounding factors

Several other factors may explain these results. For one, since we are using raw estimates of ω for correlations, if piRNA genes tend to be longer, there will be less error in their estimated values and thus more power to reveal underlying genome-wide effects. Likewise, interpretation of ω estimates can be confounded by GC content [40,41]. To tease these factors apart, we performed a multiple linear regression to determine which variables were contributing to the TE-by-ω interaction seen in the piRNA machinery but not in the control genes. Phylogenetic non-independence was accounted for by analyzing independent contrast values within a multiple linear regression framework that specified contrast ω as a function of contrast TE content, GC content at third positions (GC3) and protein length. In addition, classification as being either a piRNA gene or a control gene served as a categorical variable designated 'gene set' (Table 5). Consistent with the observed genome-wide negative correlation between TE abundance and ω, there was a strong effect of TE content on contrast ω values (*p < 0.001*). Furthermore, a significant effect of 'gene set' was also found *(p = 0.037)*. Crucially, from this analysis, there was no significant effect of GC3 (*p = 0.377*) or gene length (*p = 0.99*) on contrast ω values.

**Table 5:** Multiple regression results for contrast values of ω transformed by the Box-Cox method

Effect	DF	DF	F Value	Pr > F
TE	1	216	24.47	**< 0.0001**

Gene Set	2	20	3.91	**0.0367**

TE X Gene Set	1	216	2.99	*0.0852*

GC3	1	216	0.78	0.3766

Length	1	216	0.00	0.9898

Using contrast values controls for phylogenetic non-independence.

After accounting for these sources of variation, a test for an effect of TE abundance that is specific to the piRNA machinery can be performed by determining if there is a significant interaction between TE abundance and 'gene set'. We detected a significant TE-by-'gene set' interaction at the 0.1 level (*p = 0.085)*. Since our previous analysis suggested that correcting for phylogeny was not always warranted, this conservative framework on contrast values leads us to conclude that evolution of piRNA machinery appears especially slowed under increasing TE abundance. While not significant at the 0.05 level, we can minimally reject that increasing TE abundance leads to a higher rate of evolution in the piRNA machinery.

### Codon bias: Selection for translational efficiency in the machinery of genome defense

**Figure 6:**
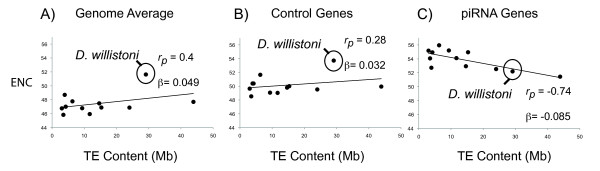
**Effective number of codons (ENC) vs. genomic TE abundance for A) genome average obtained from Vicario, Moriyama and Powell **[54]**(*p = 0.20*), B) control genes (*p = 0.364*) and C) piRNA genes (*p = 0.005*)**. *r_p_*: Pearson's correlation coefficient. β: regression coefficient (slope).

While we find no evidence of increasing rates of amino-acid evolution in the piRNA machinery under increasing TE abundance, we sought to determine whether there was any influence of TE abundance on levels of codon bias. This would be expected if increasing TE abundance demanded higher levels of protein expression for piRNA genes. First, to account for background genomic effects on codon bias, we tested whether there was a genome-wide correlation between TE abundance and codon bias as measured by the effective number of codons (ENC) (Figure 6). Across the genome, there appears to be a mild negative correlation between TE abundance and codon bias, as seen by higher ENC in species with higher TE abundance (Figure 6a, p *= 0.20; *λ test, *p = 0.0001; BayesTraits, p = 0.596*). Importantly, this is largely explained by the effect of *D. willistoni*, which is known to have a relaxed codon bias [42]. Excluding *D. willistoni*, there is only a mimimal influence of TE abundance on codon bias across the genome (*r_pearson _= 0.14*, β = 0.01). Examining only the quickly evolving control genes, the same pattern is observed (Figure 6b, p = *0.364; *λ test, *p = 0.007; BayesTraits, p = 0.556*). Thus average ENC is minimally influenced by TE abundance, with or without *D. willistoni*.

Strikingly, the opposite trend is seen in components of the piRNA machinery. There is a strong negative correlation between average ENC and TE abundance, indicating that codon bias is increased in the piRNA machinery in species with increased TE abundance (Figure 6c, p *= 0.005; *λ test, *p = 0.026; BayesTraits, p = 0.026*). This effect is also maintained within *D. willistoni*, which has high TE abundance but a genome-wide level of relaxed codon bias. To account for the effects of phylogeny and other confounding factors, we performed a separate multiple linear regression on the transformed contrast ENC values with TE abundance and gene length as predictive variables and 'gene set' as a categorical variable (Table 6). We find no significant effect of TE abundance *(p = 0.505)*, 'gene set' *(p = 0.262*) or length *(p = .955*) alone on contrast ENC values. However, there is a significant interaction between TE and 'gene set' *(p = 0.0346*), consistent with findings that TE abundance is correlated with higher codon bias specifically in the piRNA machinery. We can thus conclude that this observation is not confounded by phylogeny. Finally, it was important to determine whether codon bias and ω covaried. This might be the case if increasing codon bias was correlated with reduced ω. We considered this unlikely because the negative correlation of TE abundance and ω in the piRNA machinery was explained by dN but neither GC3 nor dS. As expected, ω was not a significant predictor of contrast ENC in the multiple linear regression analysis *(p = 0.622)*. We can thus conclude that increasing codon bias and purifying selection are separately correlated with TE abundance in the piRNA machinery.

**Table 6:** Multiple regression results for contrast values of ENC transformed by the Box-Cox method

Effect	DF	DF	F Value	Pr > F
TE	1	216	0.45	0.5048

Gene Set	2	20	1.43	0.2623

TE X Gene Set	1	216	4.52	**0.0346**

Length	1	216	0.00	0.9550

ω	1	216	0.24	0.6222

Using contrast values controls for phylogenetic non-independence.

## Discussion

Why are the genomes of some species minimally impacted by TEs but the genomes of other species greatly encumbered? Decades of work in the population biology of TEs have indicated that natural selection plays an important role in limiting their spread. However, it is not clear how variation in the strength of selection relative to genetic drift contributes to variation among species. In organisms with very large population sizes, drift is weak and modest variation in population size may play little role in determining variation in TE abundance. Conversely, drift is strong in species with small population sizes and modest variation in population size may be an important determinant of variation in TE abundance. A second important determinant of TE abundance is the machinery of piRNA-mediated genome defense. The contribution of genome defense, relative to natural selection, in constraining TE proliferation in populations is not known. Moreover, the co-evolutionary dynamics between TEs and host mechanisms of genome defense are poorly understood.

To shed light on these issues, we have looked at how patterns of molecular evolution, genome-wide and within the piRNA machinery, co-vary with TE abundance across the *Drosophila *genus. Several results were surprising. In particular, we found that genome-wide levels of purifying selection on protein-coding genes are positively correlated with TE abundance. We also found that the piRNA machinery becomes especially constrained with increasing TE content. But in the face of this constraint, we also find that the genes of the piRNA machinery show a positive correlation between codon bias and TE abundance.

### Genome-wide purifying selection on protein-coding genes and TE abundance

From this analysis we conclude that there is a robust correlation between TE abundance and the magnitude of genome-wide purifying selection on proteins. These effects are seen across different scales of divergence and with different methods for estimating ω across species. Purifying selection appears correlated with population size across the genus [43] so this is not expected under a simple model in which TE success and purifying selection on protein sequences are both solely explained by the dynamics of mildly deleterious alleles in populations of varying size. In this respect, it contrasts with the same study that found a negative relationship between the same measure of TE content and average levels of nucleotide polymorphism [43]. One cause of this discrepancy may be that polymorphism, as a measure of current population size, may influence bulk TE abundance in different ways than the historical measure ω. A similar contrast between the effects of current versus historical population size on measures of adaptation has recently been observed in *D. miranda *and *D. pseudoobscura *[44].

Our results might be explained under a population size model if a portion of non-synonymous substitutions were mildly beneficial. In this scenario TE insertions would be nearly always harmful and their abundance would largely be explained by reduced efficacy of selection in smaller populations. Under this model, increasing levels of ω in species with low TE abundance would not be driven by reduced efficacy in removing mildly deleterious non-synonymous mutations. Rather, they would be driven by an increasing rate of adaptive substitution within those populations.

While plausible, this scenario is unlikely to explain these results for three reasons. First, the rate of adaptive substitution does not seem to vary strongly across the genus [15,16]. Second, we find no significant correlation between TE abundance and genome-wide codon bias, also consistent with a previous study that found no significant relationship between genome-wide ENC and genome-wide polymorphism [43]. This suggests that across the genus species with low TE abundance have not experienced an increase in the strength of natural selection favoring mildly beneficial alleles such as favored codons (But see [45] regarding shorter time scales of divergence). Third, this scenario predicts that populations with small sizes would have higher TE abundance and this is not the case. For example, *D. grimshawi *and *D. sechellia *are both island endemics with small populations but very low TE abundance. *D. grimshawi *is endemic to the Hawaiian islands. *D. sechellia *is endemic to the Seychelles archipelago and has a population size estimated to be at least 10-fold lower than *D. simulans *[46]. In contrast, the two species with the highest TE abundance, *D. ananassae *and *D. willistoni *have broad distributions and large population sizes. *D. ananassae *has the greatest TE abundance of all twelve species and is widely distributed, with a population diversity estimate measured by *θ*_*w *_on the order of 0.009 [47]. This is similar to that observed in *D. melanogaster *(European population *θ*_*w *_: 0.006, African population *θ*_*w*_: 0.013) [48], a species with low TE abundance. Likewise, in *D. willistoni θ*_*w *_= 0.012 [49]. Overall, population size does not appear to be a good predictor of TE abundance within the *Drosophila *genus. Rather, TE success is more likely to be explained by other factors.

One possibility is that increasing population size in *Drosophila *instead leads to a greater rate of TE exposure. If TE proliferation rates are only weakly affected by population size, but population exposure rates scale with the population size, we would expect TE abundance to be partially determined in a manner opposite to the standard prediction - within the genus, larger populations with increased purifying selection on protein-coding sequences should have higher TE abundance since these populations are a larger target for TE introduction. If TE exposure rates largely determine the abundance of TEs within the genome, we would expect that species with higher TE abundance also have a higher diversity of different TE families, rather than simply having increased copy number for a similar number of TE families. This is in fact the case; we found that the number of different TE families within each species genome is a strong predictor of total TE abundance. In fact, *D. grimshawi *has only nine identified TE families whereas *D. ananassae *has 197. This supports the suggestion that reduced levels of exposure, as might be experienced by island endemics, has a strong influence on aggregate TE abundance [34]. This is also consistent with the observation that colonization of new environments by *Drosophila melanogaster *seems to have coincided with proliferation of LTR elements, perhaps due to increased exposure [50].

### Purifying selection on the piRNA machinery

In addition to the genome-wide correlation between constraint on protein-coding sequences and TE abundance, we find that this trend surprisingly also holds for the piRNA machinery itself. In fact, the effect is more apparent in the piRNA machinery in three principle ways. First, across two different scales of divergence, the distribution of correlation coefficients is significantly more negative in piRNA genes compared to control genes. Second, piRNA genes are more likely to have a significant relationship between TE abundance and ω than control genes after accounting for phylogeny. Finally, there is a marginally significant TE by 'gene set' interaction that explains variation in contrast values of ω. In several species of *Drosophila*, the piRNA machinery has been shown to evolve under a high rate of adaptive evolution. Under an arms race model of co-evolution, one might expect that an increasing TE abundance would facilitate an even higher rate of evolution in the piRNA machinery. Instead, an increasing level of purifying selection in the piRNA machinery in species with high TE abundance is evident.

There are several possible explanations for this result. Clearly, the background genome-wide effect of TE abundance on ω contributes. In fact, there may be no direct causal relationship between TE abundance and patterns of constraint on the coding sequences of the piRNA machinery distinct from the background effect. Rather, ω in genes of the piRNA machinery may simply be more susceptible to the underlying demographic factors that also explain the genome-wide correlation.

However, we cannot rule out the influence of a causative relationship between TE abundance and enhanced purifying selection in the piRNA machinery. If so, the nature of co-evolutionary dynamics make it difficult to determine whether increased TE abundance would be a cause or consequence of slower evolution of the piRNA machinery. In particular, higher TE abundance may be driven by increasing pleiotropic constraint reducing the rate of adaptation in the piRNA machinery. Species with a higher rate of evolution in the piRNA machinery may have fewer TEs precisely because the piRNA machinery is more capable of adaptation. While the primary function of the piRNA machinery appears to be the control of transposable elements, piRNAs have been found that function in gene regulation by targeting the 3' UTRs of endogenous genes [51]. Constraint on the piRNA machinery may be greater in species where this function is more critical. This increased constraint may in turn limit the ability of the piRNA machinery to adapt under an arms race model, allowing greater TE proliferation. Pleiotropic costs can act against the fixation of defense strategies [52,53] and there may be varying degrees of pleiotropy constraining the piRNA machinery across the *Drosophila *genus. This would indicate that pleiotropic constraint on genome defense could be a factor contributing to variation in TE abundance across species.

Alternatively, TE abundance and diversity may also contribute to increased conservation of the piRNA machinery. This might be the case if genomic TE abundance were driven by extrinsic factors such as exposure to vectors of horizontal TE transmission. Species with low TE exposure and burden might show a high rate of evolution in the piRNA machinery due to the combined effects of adaptive evolution, as previous studies have shown, and reduced purifying selection. On the other hand, increased TE exposure would drive a slower rate of evolution of the piRNA machinery due to the challenge of warding off multiple TE families at the same time. In contrast to species with few TEs, the piRNA machinery in species with greater TE abundance would have a reduced rate of evolution if fewer substitutions were effectively neutral or if the fixation of adaptive alleles that confer a benefit against one TE family came at the cost of defense against another family.

Under any of these scenarios, these results do not indicate that evolutionary arms races have been abetted in species with higher TE abundance. Because this study focuses on estimates of ω across whole genes, it does not account for potential variation in ω values along the gene. Therefore, while we find that the overall rate of evolution is reduced in the piRNA machinery of species with higher TE abundance, it is conceivable that a much higher proportion of non-synonymous substitutions are adaptive in species with high TE abundance. The conclusion of this study is that at whatever tempo the arms race may be occurring, increasing TE abundance is not sufficient to drive a higher rate of overall amino-acid evolution in the piRNA machinery and may even constrain adaptation of the piRNA machinery.

### Codon bias in piRNA machinery and TE abundance

If an increased rate of amino-acid evolution in the piRNA machinery is not apparent in species with greater TE abundance, might the piRNA machinery respond in alternative ways? This study indicates that an important response is increased levels of codon bias in the piRNA machinery. This effect is distinct from the effect on ω for several reasons. First, increased codon bias reduces dS and thus would act in the opposite direction as the observed decrease of ω with TE abundance. Second, multiple linear regression shows that ω is not a significant predictor of contrasts in codon bias. Third, while significant, the strength of the effect of TE content on ENC in the piRNA machinery is mild, thus having limited effect on dS. Overall, it appears most likely that codon bias in the piRNA machinery has a distinct relationship with TE content. This suggests that increasing TE abundance has selected for increased expression of proteins that comprise the piRNA machinery, rather than substantial changes in the piRNA machinery itself.

## Conclusion

We can conclude that a decrease in the historical strength of natural selection relative to drift appears unlikely to explain higher TE abundance across the genus *Drosophila*. In addition, we can conclude that increasing TE abundance does not drive a faster rate of evolution in the piRNA machinery in the *Drosophila *genus. If anything, the rate of evolution is slower. Future studies that employ denser taxon and population sampling will determine whether this is due to species specific differences in the proportion of sites undergoing adaptive or purifying evolution. Strikingly, selection under high TE abundance seems to act primarily by increasing the translational efficiency of the piRNA machinery through greater levels of codon bias. Future studies will be needed to determine whether there is a corresponding increase of protein levels of the piRNA machinery in response to greater TE abundance.

While there is clear evidence for a high rate of adaptive evolution in the piRNA machinery in some species of *Drosophila*, evolutionary arms races are not the only outcome of host-pathogen interactions. An alternative outcome may be trench warfare which occurs when a diversity of parasites selects for the maintenance of multiple defense strategies, thus tempering the rate of adaptive fixation [32]. The key signature of this alternative outcome is a signature of balancing selection. Future evolutionary studies of the piRNA machinery under varying TE load should yield great insight into the co-evolutionary dynamics that occur between TEs and their hosts.

## Methods

### Genome Wide Estimates of TE abundance, ω and Codon Bias

Previous estimates of TE abundance, ω and codon bias were used for genome wide calculations. Genomic TE abundance was estimated as the product of the percent TE/Repeat in the assembled genome and the assembled genome size as reported by the 12 Genomes Consortium, ranging from 3% to 24% of euchromatic content in these species [34]. This provides a measure of bulk DNA content comprised of TE/repeat sequences in the assembled sequence, rather than copy number. We chose this method to capture the measure of TE abundance that is most likely to influence host fitness. Ectopic recombination and the metabolic costs of TE replication, transcription and translation are important factors that contribute to selection against TEs and these will scale with bulk TE/repeat content in assembled euchromatin. Moreover, while copy number might serve as a better approximate measure of the fitness affects due to insertional mutation, since the distribution of TE element classes does not widely vary among species [34], bulk repeat content serves as a good proxy of copy number.

Genome wide estimates of ω were obtained from the analysis of Heger and Ponting [33] who used a set of 6375 orthologous gene sets. In their analysis, terminal branch ω estimates were obtained using a model of PAML [38] that allowed branch-specific ω estimation, excluding *D. sechellia *, *D. persimilis *and *D. willistoni*. Genome wide estimates of codon bias, as measured by the effective number of codons (ENC) were obtained from the analysis of Vicario, Moriyama and Powell [54].

### piRNA and control gene selection

piRNA genes were defined as genes that code for proteins involved in piRNA biogenesis and/or composition of the nuage. These were: *Argonaute 3*, *armitage*, *aubergine*, *cutoff*, *krimper*, *maelstrom*, *piwi*, *spindle-E*, *squash*, *vasa*, and *zucchini*. Rhino is a protein that is involved in piRNA biogenesis but no suitable alignments could be generated across the genus. For this reason, *rhino *was not included in the analysis. Alignments were downloaded from the UCSC Genome Browser http://hgdownload.cse.ucsc.edu/goldenPath/dm3/multiz15way/alignments/flyBaseGene.exonNuc.fa.gz and were manually edited to remove ambiguous bases and incomplete codons to maintain reading frame. We also selected a set of control genes that were similar in the distribution of ω values in aggregate across the *Drosophila *phylogeny. This was done to minimize statistical artifacts that might arise if the mean and variances in the distribution of ω values differed strongly between the two sets, especially since piRNA genes are already known to evolve quickly (see analysis of ω below). Moreover, if piRNA genes also have more variance in ω than the average protein coding genes, using a set of genes selected based on mean ω value alone may introduce bias by favoring the detection of an effect of TE abundance on piRNA genes relative to other genes. To deal with this, a control set of comparison genes was selected with similar mean and variance for ω. Initially, a total of 30 genes with previously estimated ω values estimated on the *D. melanogaster *subgroup [34]ftp://ftp.flybase.net/genomes/12_species_analysis/clark_eisen/paml/ were selected based on similarity of previously calculated ω values to those from piRNA genes. Since piRNA genes have already been shown to have signatures of faster evolution, we attempted to avoid polluting this control set of faster evolving genes with other unknown piRNA genes by requiring genes in the control set to have a biological function not one involved in an RNA mediated process, as indicated in the Flybase gene summary http://www.flybase.org. Path ω values from the UCSC Genome Browser alignments were estimated for each of the 30 initial genes using the codeml free-ratio branch model as with the piRNA genes (see below). Rather than simply matching based on mean ω, the control ω distributions for each gene (without regard to species) were then iteratively matched to piRNA gene ω distributions by completing all pairwise comparisons using Cramér Von-Mises goodness of fit tests. The piRNA-control gene pair that that returned the largest p-value from the Cramér Von-Mises test was considered the best match. If two piRNA genes had the same control gene as their best match the p-values of the Cramér Von-Mises tests were compared and the gene pair with the larger p-value was retained and the next best match was assigned as the pair for the other gene, until all the piRNA genes were matched with a control gene. This method acted to make the distribution (among genes) of ω distributions of the piRNA and control sets similar. The final control set for chosen for the twelve species analysis was: CG8222, CG7876, CG2595, CG31605, CG31287, CG33934, CG6898, CG15013, CG4815, CG11405, CG4259.

A second control set was chosen for the *D. melanogaster *subgroup (*D. melanogaster, D. sechellia, D. simulans, D. yakuba, D. erecta*) through the same iterative procedure, using the original sample of 30 genes. This was performed because the distribution of ω values differs between the entire 12 species phylogeny and the *D. melanogaster *subgroup. The selected control set for this group was: CG8348, CG10372, CG8222, CG6898, CG2595, CG33934, CG4815, CG31605, CG30169, CG4259, CG31287.

### ω Estimates Along Evolutionary Paths

ω was estimated using codeml implemented in PAML v 4.3 [38] with a free-ratio branch model. We specifically used branch models to evaluate ω along evolutionary paths (denoted the foreground) against the rest of the phylogeny (denoted the background) for each gene and each species in the phylogeny. We define an evolutionary path as all the branches across a phylogeny that connect a given extant taxon to common ancestor located at a particular node. The first set of analyses was informed by a phylogeny containing the 12 species of *Drosophila *with sequenced genomes based on the topology reported by the 12 genomes consortium [34]. For each species, ω was estimated on the branch descendant from either the node that defines the *D. virilis, D. mojavensis *and *D. grimshawi *clade or the node that defines the clade of remaining species. The second set of analyses focused exclusively on the 5 species of the *D. melanogaster *subgroup and was informed by a phylogeny of the *D. melanogaster *subgroup pruned from the 12 genomes phylogeny. ω estimates were obtained for the branches connecting to either the node uniting the *D. melanogaster *and *D. simulans *complex or the node connecting the *D. erecta *and *D. yakuba *lineages. We did not include the internal branch connecting these nodes in foreground branch estimates.

In each individual path analysis the evolutionary model used estimated two ω ratios. The first ω ratio (the foreground ω ratio) described evolution along the evolutionary path of interest while the second (the background ω ratio) estimated evolution across the rest of the phylogeny. Foreground ω values were retained for subsequent analyses. This foreground/background approach was chosen for several reasons. First, path ω values were favored over tip ω values to reduce the effects of variance on estimated ω ratios on short tip branches such as those for *D. persimilis*. In this analysis, path distances are much more similar across species than tip distances. Secondly, using this approach of path ω is fairly conservative for detecting effects of TE abundance. This is because much of the estimate for path ω for a given species is informed by internal branches whereas the estimates for TE abundance are tip estimates. The effect of this will be to dampen signal that arises from any potential correlation between natural selection acting in present time on piRNA genes and contemporary TE abundance. Thirdly, while ω estimates from related species will be jointly determined by shared internal branches, correcting for phylogenetic non-independence deals with this problem.

### Phylogenetic analysis of the Correlation Between ω and Transposable Element Abundance

Standard regressions between ω and Transposable Element (TE)/repeat amount were analyzed for each gene separately and statistical significance was determined using the R package. Shared ancestry causes phylogenetic non-independence and statistical analysis of correlations was also performed accounting for this using the program *Continuous*, implemented in *BayesTraits *([37] available from http://www.evolution.rdg.ac.uk.). We did not determine whether phylogenetic correction was necessary for analyses involving the *D. melanogaster *subgroup due to the inability to generate stable likelihood scores arising from the parameter richness (compared to the number of taxa) of the models. We feel that this simplification for the *D. melanogaster *subgroup is acceptable due to low power of comparative methods when taxon sampling is low [39].

Treating both TE amount and ω as continuous traits, the *BayesTraits *implementation of *Continuous *was used to model the phylogenetic influence on these traits using a generalized least-squares approach that assumes a Brownian motion model of evolution [37]. Likelihood scores of greater magnitude were obtained for a directional random-walk model compared to a constant-variance random walk model so directional models were employed. Directional models were then evaluated in a likelihood-ratio framework by comparing nested models that differed by the inclusion of the scaling parameter λ, which reveals whether the phylogeny correctly predicts the patterns of variance among species for a single trait and, in the case of two traits, for both simultaneously. In the first model λ was freely estimated, and in the second model λ = 0. A λ value of zero models trait evolution on a star phylogeny and signifies no phylogenetic correction. The likelihood scores of the two models for each gene were compared using likelihood ratios (LR). If the LR was not significant then phylogenetic correction was unnecessary. For these correlations the Pearson's correlation coefficient was calculated and evaluated for significance in R. If the LR was significant then we estimated the significance of the correlation between the traits using phylogenetically independent contrasts [36].

Since trait values for species are contingent on phylogenetic history, species trait values are not independent. However, changes in trait values that occur on different lineages are. Thus, rather than looking at the correlation between species trait values, one examines the correlation between evolutionary changes in trait values inferred on the phylogeny. This is known as phylogenetically independent contrasts analysis, where a contrast refers to the inferred trait changes that have occurred along a focal lineage. Using contrast values, standard statistical procedures can be applied. For our independent contrast analyses we used *Continuous *in *BayesTraits*. Several scaling factors are used in *Continuous *to parameterize the model of evolution of the trait on the phylogeny. When each of these parameters is set to one, the tree topology and branch lengths are assumed to accurately describe the evolution of the trait. Setting each of the scaling parameters to one provides results equivalent to standard independent contrast analyses (*Continuous *manual, http://www.evolution.rdg.ac.uk). To test for a significant correlation using this method, we compared likelihood scores in which the covariance between TE amount and ω was freely estimated and in the second model the covariance = 0. We compared likelihood scores of the two models for each gene using LRs. If the LR of the comparison was significant, we concluded there was a significant correlation between the traits after correcting for phylogenetic relationships. Correlations between dS and TE amount and dN and TE amount were also analyzed in the same manner described above.

For this analysis tree topology and branch lengths were kindly provided by Patrick O'Grady and Sudhir Kumar:

(((((dsim:0.02120277, dsec:0.02358723):0.02898348, dmel:0.05989652):0.06713426,(dyak:0.09667234, dere:0.08942766):0.03200292):0.43736490, dana:0.60757469):0.11949656,(dpse:0.00957250, dper:0.01847750):0.51906573,(dwil:0.69140554,(dgri:0.39516469,(dmoj:0.38155611, dvir:0.33379389):0.06304531):0.25381446):0.08257927);

### Additional statistical analysis

ENC was determined using CodonW. 95% C.I. for the estimate of mean correlation coefficient were computed with 10,000 bootstrap estimates of the mean. Tests for the difference in mean correlation coefficient were determined by estimating 10,000 bootstrap comparisons in the mean and determining the probability that the control means were less than or equal to the piRNA means. This approach was used due to the violation of normality for correlation coefficients that are between -1 and 1.

While *Continuous *in *BayesTraits *can test for correlations between two traits, it is difficult to examine the relationship between multiple different traits. Since other variables could confound these results, we sought to determine whether other variables such as gene length and GC content at 3^rd ^positions were significant predictor variables of contrast ω that could explain the results reported. To do this, we performed multiple linear regression on phylogenetically independent contrast values estimated using the ape package in R. Before contrasts were estimated variables were transformed as necessary with power transformations to meet normality assumptions of phylogenetically independent contrast methods. The power of the transformation was determined using the Box-Cox method. Contrast values were then used in multiple linear regression with contrast ω the dependent variable and contrast TE abundance, gene length, and GC content as independent variables. Gene function (piRNA or control) was treated as a categorical variable. A similar framework was used for ENC. The coefficients of the linear models were computed using Maximum Likelihood in SAS with the PROC MIXED procedure. Even though we did not report mixed models in this study we used PROC MIXED during the model selection process and thus used this procedure for reduced models as the results are equivalent to standard linear regression procedures.

## Authors' contributions

JPB conceived the study. DCM performed the primary analysis and method development. JPB performed additional analysis. JCM and KSB assisted in the analysis. JPB, DCM and JCM drafted the manuscript. All authors read and approved the final manuscript.
